# Psychological stress, the central nervous system and arrhythmias

**DOI:** 10.1093/qjmed/hcad144

**Published:** 2023-07-05

**Authors:** P D Lambiase, S N Garfinkel, P Taggart

**Affiliations:** UCL Institute of Cardiovascular Science & Barts Heart Centre, Rayne Institute, 5 University Street, London WC1E 6JF, UK; UCL Institute of Cognitive Neuroscience; UCL Institute of Cardiovascular Science & Barts Heart Centre, Rayne Institute, 5 University Street, London WC1E 6JF, UK

## Abstract

This review highlights the links between psychological stress and the neurocircuitry of cardiac–brain interactions leading to arrhythmias. The role of efferent and afferent connections in the heart–brain axis is considered, with the mechanisms by which emotional responses promote arrhythmias illustrated by inherited cardiac conditions. Novel therapeutic targets for intervention in the autonomic nervous system are considered.

## Evidence for psychological stress as a cause of arrhythmias and sudden cardiac death

**Figure 1.**
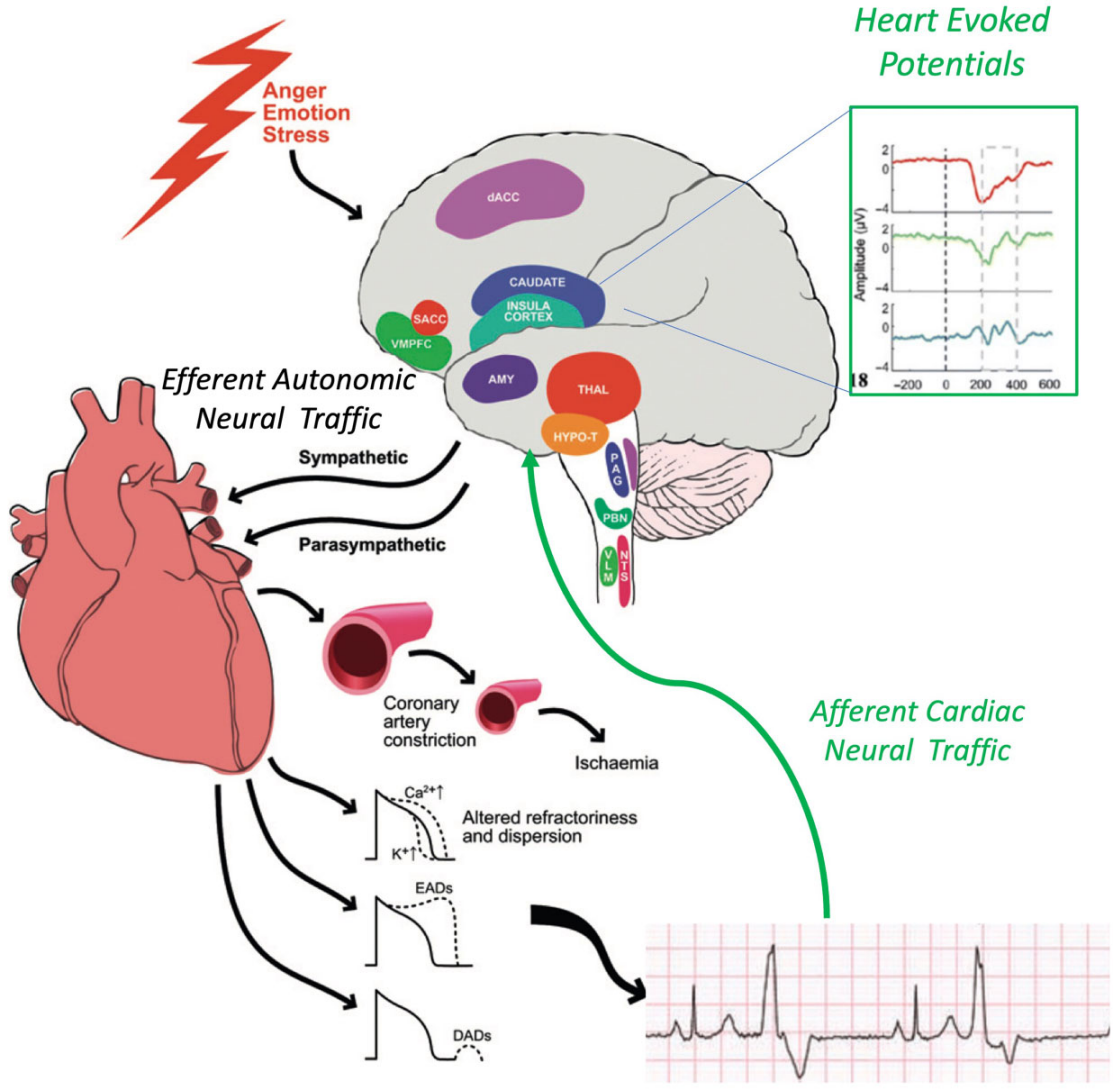
Schematic diagram illustrating effects of mental stress on the CNS and efferent autonomic traffic modulating cardiac electrical activity. An afferent limb of neural feedback from the heart, operating through a number of pathways relayed up through cranial nerves and spinal relays to cortical and deep structures, including the amygdala, the region of the ventral anterior cingulate cortex–ventral anterior prefrontal cortex (vACC–vmPFC), the insula and the somatosensory cortex (SS cortex), plays an important role in the integration of these signals. HEPs can then be recorded in this region just after the R-wave on the ECG which could also modulate forward neural traffic to the heart—a subject of ongoing investigation (adapted from Ref. 31).

**Figure 2.**
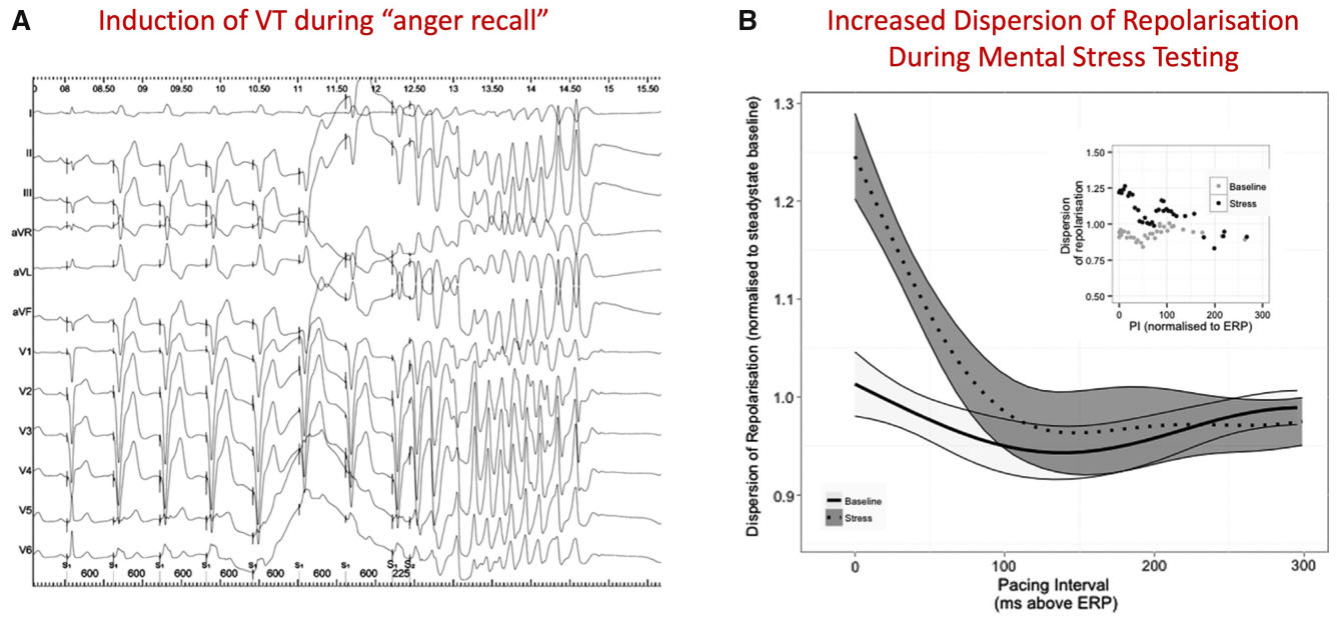
(**A**) Induction of VT during an anger recall experiment which was not inducible at rest. (**B**) Dispersion of repolarization. The dynamics of total dispersion of repolarization obtained in 12 patients are shown, normalized to mean dispersion during steady-state pacing during active relaxation. Pacing interval (PI) is normalized to milliseconds above the effective refractory period (ERP). The shaded bands represent 95% confidence intervals, calculated using LOESS (LOcal regrESSion) regression, and separation of these bands implies statistical significance. There is a significant increase in the dispersion of repolarization during stress as pacing intervals approach effective refractory period during stress, which is not present at rest (*P* < 0.001)—adapted from Ref. 4.

The literature abounds throughout the ages with anecdotal cases of sudden death following a mentally stressful occurrence. A much-quoted example is that of the surgeon John Hunter, who predicted that his death might occur if he was aroused to anger. This indeed subsequently proved to be the case in a hospital board meeting. While anger appears to be the emotion most commonly associated with sudden cardiac death (SCD), a range of other emotions have been implicated. There is now substantial evidence to support the notion that mental stress may play a major role in SCD. For example, the incidence of SCD increases in the days following national disasters, such as earthquakes.^1^^,^^2^ Experimental work in animal models has shown that stress amplifies the pro-arrhythmic effects of ischaemia.^3^ In humans, anger has also been shown to potentiate ventricular arrhythmias (Figure 1). Mental stress may induce coronary artery or microvascular constriction and cause angina in some patients. A variety of mental stress protocols produce electrophysiological changes relevant to arrhythmogenesis, such as changes in the ECG T wave, T-wave alternans and alteration in ventricular action potential repolarization, which set up the conditions for ventricular arrhythmias (Figure 2). Indeed, we have demonstrated such effects in the cardiac catheterization lab with invasive recordings (Figure 2).^4^ Emotion may precipitate ventricular fibrillation in channelopathy syndrome patients [long QT syndrome (LQTS) and catecholaminergic polymorphic ventricular tachycardia (CPVT), see below].

## Heart–brain interactions

### Emotions

#### Neural circuitry, autonomics and interoception

The heart and brain are dynamically coupled; the strength and timing of each heartbeat are communicated to the brain via the activation of arterial baroreceptors located in the aortic arch and carotid sinuses. These phasic signals elicited at each heartbeat shape the representation of cardiovascular arousal throughout the brain. The initial encoding of this afferent information within brainstem centres is relayed onwards to the basal ganglia and amygdala, and through thalamocortical projections to the insular, orbitofrontal and anterior circulate cortices.^5^ These neural areas are also involved in emotional and motivational behaviour; the body and brain are integrated to inform emotional feeling states and motivational drive influenced by changes in cardiovascular state.

An early PET study^6^ provides compelling evidence for understanding cardiac afferent signalling. Patients with (non-diabetic) coronary artery disease were given low-dose dobutamine to induce mild ischaemic stress. ECG evidence of ischaemia was associated with increased activity (regional cerebral blood flow) to thalamus and right frontal cortex. Those that additionally experienced angina showed heightened activation within basal frontal, temporal and ventral cingulate cortices. These findings suggest that perceptual feeling states induced by (normal and abnormal) cardiac afferent signalling are supported by cortical engagement (with insula particularly highlighted as important). Moreover, they indicate a hierarchical representation within the brain, above the level of the medullary baroreflex, of the integrity of cardiac function. The baroreflex itself is a key hub in supporting afferent–efferent interactions in the autonomic control of the heart. However, ascending cardiac feedback is coupled to descending autonomic drive at multiple levels of the neuroaxis, consistent with explanatory models of predictive coding and active inference.

Indeed, the cortical representation of heartbeats can be measured using techniques such as scalp electroencephalography (EEG) to extract the heartbeat-evoked potential (HEP), which is an event-related potential time-locked to participants’ heartbeats. The amplitude of the HEP corresponds to the magnitude of the cortical processing of individual heartbeats. In a combined cardiac and EEG study of mental stress, HEP amplitude at left temporal and lateral frontal electrode locations correlated with stress-induced changes in cardiac output, consistent with an afferent cortical representation of myocardial function during stress. Moreover, the amplitude of the HEP in the left temporal region reflected the proarrhythmic status of the heart (inhomogeneity of left ventricular repolarization).^7^ These observations delineate a cortical representation of cardiac function predictive of proarrhythmic abnormalities in cardiac repolarization.

The HEP signal is altered in individuals who have dispositional changes in emotion processing, such as those with borderline personality disorder where HEP amplitudes are negatively correlated with emotional dysregulation in this population.^8^ High-resolution functional neuroimaging combined with physiological recordings during emotional processing reveals the central representation of cardiac signal in the posterior insula. Specifically, activity within the posterior insula is coupled to cardiac changes, including the high-frequency component of heart-rate variability.^9^ In contrast, accurate perception of cardiac signals is represented in the right anterior insula.^10^ Neural signatures of body–brain integration, such as the HEP, offer state-of-the-art techniques providing novel quantitative insight into the cerebral representation of visceral information. This is a meaningful index for a range of processing, with preliminary research using evoked pain in healthy individuals suggesting that increased levels of body–brain integration are associated with a higher threshold for pain, and that HEP amplitude is inversely related to pain ratings.^11^ Moreover, hypertensive patients show decreased sensitivity to pain and higher HEP amplitude. Chronic pain is associated with increased risk of hypertension, consistent with a dysfunctional interaction between cardiovascular and pain modulatory systems. This evidence potentially links pain states to altered cortical measures of heart–brain integration.

Interoception is defined as the process by which the nervous system senses, interprets and integrates signals originating from within the body, providing a moment-by-moment mapping of the body’s internal landscape across conscious and unconscious levels. Contemporary models of interoception include the nature of afferent signals, as well as their neural and higher order processing.^12^ Individuals differ in their capacity to accurately sense internal bodily signals, and these differences in interoceptive accuracy map onto behaviours which are informed by internal bodily signals, such as emotion processing^13^ as well as extent of neural activity within the anterior insula during a heartbeat perception test. Individual differences in interoceptive accuracy are also related to decision making guided by internal bodily signals, such as ‘gut instinct’, a form of intuitive decision making that can be mapped onto different cardiovascular responses.^14^

## Mental stress and arrhythmia

Stress-induced excessive beta-adrenergic stimulation, by increasing the balance of inward relative to outward currents across the cell membrane, may generate spontaneous depolarizations (after-depolarizations) manifest as ectopic beats or if repetitive manifest as focal tachycardia. Mental stress may enhance action potential duration (APD) inhomogeneity and generate a disordered pattern of refractoriness and excitability. This may destabilize the activation wavefronts and increase susceptibility to re-entrant arrhythmias (Figure 1).

Pathological processes such as ischaemia, myocardial infarction and cellular and neural remodelling can alter and magnify the electrophysiological response to sympathetic stimulation and are themselves characteristically inhomogeneous. Patients with genetic dysfunction of ion channels—‘channelopathies’—may be especially sensitive to potentially pro-arrhythmic effects of mental stress as discussed below. Another potential mechanism may be relevant to the effect of abrupt stress, such as a sudden fright. Prolonged beta-adrenergic stimulation shortens APD. However, recent studies in myocytes and modelling have demonstrated a biphasic response of ventricular APD in the immediate few beats following abrupt stimulation. This biphasic response was the result of a mismatch between the fast phosphorylation/dephosphorylation time constants of the L-type calcium current (I_CaL_) which tends to prolong APD, and the slower time constants of slow component of the delayed rectifier current (I_Ks_) which shortens APD. This may enhance local repolarization inhomogeneity and can be relevant to SCD occurring immediately following a sudden shock (LQTS).

Another proposed mechanism known as the laterality hypothesis suggests that differential left/right hemisphere contributions to the processing of stress and negative emotions may generate an inhomogeneous sympathetic neural input to the heart and so be proarrhythmic.^15^ This hypothesis is supported by the known ‘ipsilateral channelling’ of neural activity through brainstem regions and the selective distribution of the right- and left-sided sympathetic nerves to the anterior and inferior/posterior regions of the heart, respectively. Such a mechanism has been proposed as a link between cardiac arrhythmias associated with focal epilepsy and stroke. Indeed, the right insular has been implicated in sudden death—in one study, five (of 62) patients with right insular infarction experienced sudden death compared with two sudden deaths in patients with left insular infarction.^9^^,^^16^^,^^17^

## Dynamic interactive substrate

The majority of serious ventricular arrhythmias and ventricular fibrillation (VF) arrests occur in people with cardiac pathology, usually coronary artery disease. This requires a trigger and the presence of a substrate such as hypertrophy, fibrosis, an anatomical scar and ischaemia. However, recent studies highlight the dynamic nature of this substrate due to constantly changing electrophysiological properties that partly reflect fluctuations in autonomic nerve balance. For example, sympathetic nerve activity strongly influences major components of arrhythmogenesis, namely automaticity, after-depolarizations, ventricular and atrial APD and refractoriness, APD restitution, conduction velocity and dispersion of repolarization.^18–20^ Sympathetic effects on repolarization and refractoriness are magnified in the presence of ischaemia. The magnitude of beat-to-beat repolarization (T-wave) alternans, a common precursor of VF, is enhanced by increased sympathetic activity and reduced by β-blockade. This variability in repolarization creates the optimal milieu for lethal arrhythmia in the substrate. However, in addition to this, a trigger such as a ventricular ectopic beat is necessary to initiate ventricular tachycardia (VT) or VF. Such triggers have been described in idiopathic VF and may be eliminated through ablation if consistently arising from the Purkinje network. Autonomic activity influences the development of these triggers.

## Genetic predisposition

A number of inherited conditions predispose to lethal ventricular arrhythmias under conditions of increased mental and adrenergic stress. The most well recognized are ion channelopathies including LQTS and CPVT.^21^^,^^22^ These conditions are directly influenced by the autonomic nervous system. Furthermore, there is an emerging literature relating the gene–environment interactions determining the neural patterning responsible for the stress response itself. Heritability accounts for 30–40% of the variance contributing to risk for mood and anxiety disorders as well as post-traumatic stress disorder. Childhood exposure to abuse and other early life adverse events increases risk for the later development of these conditions. A persistent finding in patients with depression is the elevation in the numbers of corticotrophin-releasing hormone (CRH) and arginine-vasopressin neurones in the paraventricular nucleus (PVN) in comparison to control subjects.^23^ Genetically modified lentiviral vectors were introduced into the central nucleus of the amygdala (CeA) resulting in overexpression of CRH and arginine-vasopressin within the CeA as well as the PVN in a rat study. Overexpression of CRH in these structures was accompanied physiologically by decreased glucocorticoid negative feedback, and behaviourally by increased anxiety-like behaviour (acoustic startle) and depressive-like behaviour (forced swim). These data suggest that unrestrained CRH synthesis in the CeA may produce dysregulation of the hypothalamic–pituitary–adrenal axis, which is associated with many of the behavioural, physiological and reproductive consequences associated with stress-related disorders. Furthermore, gene association studies implicate certain CRHR1 polymorphisms with depression and suicidality. This indicates that there are genetic influences determining the neurobiology of behavioural and emotional responses as well as the more precisely characterized genetic determinants of myocardial electrophysiological responses to emotional stress. Indeed, CRH also promotes atherosclerosis to create the coronary triggers for ischaemic arrhythmias.^24^

## Increased sympathetic drive is pro-arrhythmic in LQTS types 1 and 2

SCD is more common under conditions of increased anxiety (and during exercise) in these two subtypes of LQTS. Enhanced sympathetic activity can substantially increase spontaneous inward current through L-type calcium ion channels to increase the likelihood of early afterdepolarisations (EAD).^21^ Clinical data indicate that carriers of mutations in either KCNQ1 or KCNE1 are at increased risk of experiencing fatal arrhythmias in the context of elevated sympathetic activity. In long QT subtypes 1 and 2, increased adrenergic tone prolongs the QT interval at peak exercise and in early recovery. This is opposite to the normal response of QT shortening on exercise and reduced myocardial repolarization times in conditions of increased adrenergic stress. Genotype can also influence risk, with mutations for long QT 1 and 2 affecting the pore-forming region carrying the highest risk. In long QT 1, a reduced gating frequency of the slow component of the delayed rectifier potassium current (Iks) during conditions of increased adrenergic tone may explain the failure of APD and QTc to shorten during exercise and thus promote regional heterogeneities in APD and thus VT/VF. Another link between LQTS and the sympathetic nervous system is disclosed by a recent study showing that ERG channel genes are expressed in chromaffin cells, especially adrenaline-containing cells, and sustain a potassium ion current.^25^ Blockers of ERG channels modify the excitability of single chromaffin cells and increase the release of catecholamine-amplifying repolarization changes in long QT under conditions of increased emotional stress.

Schwartz *et al.* demonstrated that baroreceptor sensitivity and heart rate variability predict a higher risk of clinical events in a certain form of long QT 1 due to the expression of two polymorphisms that enhance adrenergic activity. The ADRA2C-Del322-325 polymorphism causes reduced function of an α2 adrenergic receptor, which in turn enhances norepinephrine release by reducing inhibitory feedback.^21^^,^^25^^,^^26^ The ADRB1-G389R polymorphism enhances coupling of the β1 adrenergic receptor to adenylate cyclase and augments adrenergic stimulation. Correlations between these polymorphisms and baroreceptor sensitivity are intriguing and support the inference that higher baroreceptor sensitivity reflects higher sympathetic tone. Variations in autonomic tone can thus directly affect myocardial electrophysiology in LQTS and these may be exaggerated by changes in emotional state. Indeed, high baroreceptor sensitivity is pro-arrhythmic in LQTS as it promotes more dynamic changes and decelerations in heart rate leading to early after-depolarizations and triggered events.

CPVT is an autosomal dominant condition associated with increased sarcoplasmic reticulum calcium leak via the ryanodine receptor or impaired calcium binding in the SR due to abnormalities in calsequestran in the autosomal recessive form. It presents with bidirectional VT (alternating axis ectopic beats/VT) during exercise when the heart rate reaches 120 beats per minute. Both CPVT and Long QT 1 and 2 are treated with beta blockers with centrally acting non-specific agents such as nadolol proving the most efficacious. However, in resistant cases, there is good evidence supporting the use of stellectomy targeting the lower half of the left T1 ganglion and T2–4 ganglia supplying the heart.

This approach now highlights the possibility of utilizing interventions on the autonomic nervous system directly to inhibit arrhythmias.

### Autonomic nervous system interventional targets

In the context of ventricular arrhythmias, not only is stellectomy effective in ion channelopathies but can be utilized in patients with VT storm post myocardial infarction. This is thought to relate to infarct borderzone sympathetic nerve fibres triggering ectopy and cardiac arrest due to aberrant remodelling.

This autonomic target opens the window to utilize optogenetic or device-based strategies to modulate autonomic tone targeting the stellate or vagal nuclei in the brainstem. One approach targeted the left stellate ganglion (LSG) using optogenetics to overexpress channel ArchT, an inhibitory light-sensitive opsin. This hyperpolarized the stellate cells and prevented their firing when a light was activated, preventing sympathetic traffic to the heart and inhibiting the initiation of VF in a canine model during myocardial ischaemia.^27^ Transient light-emitting diode illumination significantly suppressed the LSG function, LSG neural activity and sympathetic nerve indices of heart rate variability as well as prolonged left ventricular effective refractory period and APD90 only in the optogenetics group. Importantly, all of these changes returned to baseline within 2 h after illumination was turned off. Moreover, the ischaemia-induced VAs were significantly suppressed by illumination only in the optogenetic treated group.

Indeed, the activation of vagal nuclei in the brain utilizing an optogenetic approach has improved left ventricular remodelling post MI and could represent an alternative anti-arrhythmic target. Vagus nerve stimulation (VNS) reduces myocardial injury, while remote preconditioning (RPc) cardioprotection was found to be abolished in conditions of bilateral vagotomy.

Neurones of the brainstem dorsal motor nucleus of the vagus nerve (DVMN) were targeted using viral vectors to express a *Drosophila* allatostatin receptor (AlstR) or light-sensitive fast channel rhodopsin variant (ChIEF), respectively. RPc cardioprotection, elicited by ischaemia/reperfusion of the limbs, was abolished when DVMN neurones transduced to express AlstR were silenced by selective ligand allatostatin or in conditions of systemic muscarinic receptor blockade with atropine. In the absence of remote ischaemia/reperfusion, optogenetic activation of DVMN neurones transduced to express ChIEF reduced infarct size, mimicking the effect of RPc. This indicates that counterbalancing increased sympathetic tone with activation of the vagus has cardio-protective and potentially anti-arrhythmic effects.^28^

Tragus nerve stimulation has been shown to reduce atrial fibrillation in human studies. VNS is approved for the treatment of drug-refractory epilepsy. Transcutaneous VNS, by stimulating the auricular branch of the vagus nerve at the tragus of the external ear, is an emerging non-invasive alternative to VNS. Functional cardiac magnetic resonance studies illustrate that central projections of the vagus nerve in the brain stem and other higher centres in the brain are activated. Tragus stimulation decreases sympathetic tone in humans and suppresses inflammatory cytokines.^29^ Several studies have shown that autonomic neuromodulation with low-level VNS can suppress AF in experimental models. More recently, in a proof-of-concept human study, in patients with drug-refractory paroxysmal AF, low-level transcutaneous electrical stimulation of this auricular branch of the vagus nerve at the tragus of the ear (low-level tragus stimulation) for just 1 h significantly shortened AF duration and decreased inflammatory cytokines.^30^ This opens the avenue to the utilization of non-invasive vagal stimulation approaches to suppress arrhythmias and the cardiac effects of mental stress.

In conclusion, psychological stress induces arrhythmias through a combination of central and autonomic effects. With increasing understanding of the neuro-cardiac axis and the pathways involved, a number of potential therapeutic targets are emerging using cognitive or more direct neuromodulatory approaches.
